# Apolipoprotein CIII may mediate the impacts of angiopoietin-like protein 8 on triglyceride metabolism

**DOI:** 10.1186/s12944-018-0777-6

**Published:** 2018-07-18

**Authors:** Mengdie Luo, Xin Su, Yuhong Yi, Yang Yang, Daoquan Peng

**Affiliations:** Department of Cardiovascular Medicine, the Second Xiangya Hospital, Central South University, Changsha, 410011 Hunan China

**Keywords:** ANGPTL8, apoCIII, Triglyceride, Coronary artery disease

## Abstract

**Background:**

Angiopoietin-like protein 8(ANGPTL8) and apolipoprotein CIII (apoCIII) were found to inhibit the activity of lipoprotein lipase (LPL) and disrupt the clearance of triglyceride-rich lipoproteins (TRLs), leading to hypertriglyceridemia. Whether any relationship exists between these two important modulators of triglyceride metabolism has not been reported. Besides, whether ANGPTL8 concentration is altered in the patients with coronary artery disease (CAD) is still unclear.

**Methods:**

A hospital-based case-control study was conducted. Sixty-eight CAD subjects and fifty-two nonCAD controls were recruited. Plasma apoCIII, ANGPTL8 was measured.

**Results:**

ANGPTL8 and apoCIII concentration exhibited no significant difference between CAD group and nonCAD group. Both ANGPTL8 and apoCIII were significantly correlated with triglyceride level(*r* = − 0.243, *P* = 0.008; *r* = 0.335, *P* < 0.001, respectively). Regression analysis revealed that apoCIII was an independent contributor to triglyceride level independent of ANGPTL8 concentration (standardized β = 0.230, *P* < 0.01).

**Conclusion:**

ApoCIII may mediate the effects of ANGPTL8 on triglyceride metabolism.

## Background

The angiopoietin-like proteins (ANGPTL1–8) are secreted glycoproteins sharing common structure but exerting distinct physiological effects [1]. ANGPTL8, also referred to as betatrophin, lipasin, refeeding-induced in fat and liver (RIFL) and hepatocellular carcinoma-associated protein (TD26), was found to be a novel player in lipid metabolism [2]. Recent findings revealed that ANGPTL8, together with ANGPTL3 and ANGPTL4, controlled by nutritional status, could regulate triglyceride metabolism by inhibiting the activity of lipoprotein lipase (LPL) [3, 4], the rate-limiting enzyme for triglyceride hydrolysis and plasma triglyceride clearance [5]. Different researches have shown that ANGPTL8 concentrations were altered in diseases such as obesity, diabetes mellitus, metabolic syndrome [2] and non-alcoholic fatty liver disease (NAFLD) [6]. However, whether ANGPTL8 concentration is altered in the patients with coronary artery disease (CAD) has not been reported.

Liver-derived apolipoprotein CIII (apoCIII), mainly presents on the surface of triglyceride-rich lipoproteins (TRLs) and high-density lipoprotein (HDL) [7], was proved to be a crucial regulator of triglyceride metabolism [8–10]. ApoCIII could inhibit the binding of TRLs with LPL and impair TRLs clearance [7], which deteriorated lipid disorders and accelerated CAD progression. Although ANGPTL8 and apoCIII exerted similar effects on triglyceride metabolism, the relationship between these two triglyceride modulators was still unclear.

In the present study, we conducted an observational study to examine the ANGPTL8 and apoCIII level in different groups classified by the anthropometric and metabolic profiles. Besides, the relationship between ANGPTL8 and apoCIII and their effects on triglyceride metabolism were also studied.

## Methods

### Subjects

We recruited 68 CAD subjects and 52 nonCAD subjects from the Department of Cardiovascular Medicine of the Second Xiangya Hospital, Central South University. In this study, CAD patients were mainly composed of acute coronary syndrome (ACS), including ST-segment elevated myocardial infarction (STEMI), non ST-segment elevated myocardial infarction (NSTEMI) and unstable angina. ACS was diagnosed by the clinical symptoms and signs, ischemic electrocardiographic abnormalities, and coronary angiography showing ≥50% stenosis in at least one main coronary artery. The exclusion criteria included: a history of renal failure, chronic hepatic diseases, high fever, or bacterial/viral infection, autoimmune disease, arthritis, malignancies, severe diabetes and hypertension, and other severe medical illnesses.

### Clinical and biochemical measurements

Patient information, including age, gender, smoking and drinking history, and statin therapy history, was recorded. The details of anthropometric measurements (weight, height, body mass index) were assessed after overnight fasting for at least 10 h. Peripheral blood samples were obtained from patients’ brachial veins. Subjects fasted for at least 10 h before blood collection and then blood routine, urine routine, concentrations of lipid parameters, including total cholesterol (TC), triglyceride (TG), low-density lipoprotein cholesterol (LDL-C), HDL-C, apoAI, apoB, free fatty acid (FFA), were evaluated via an automated analyzer (Hitachi P7600). Concentrations of high-sensitivity C-reactive protein (hsCRP) were measured with a latex particle, enhanced immunoturbidimetric assay. For the subsequent experiments, fresh plasma was obtained by centrifugation at 3000 r/min at 4 °C for 10 min. The plasma was aliquoted and stored at − 80 °C freezer until analysis.

### Measurement of plasma apoCIII and ANGPTL8

Plasma ApoCIII and ANGPTL8 concentration were measured with commercially available ELISA kits (apoCIII: Abcam, ab154131, UK; ANGPTL8: EIAAB, E11644H, Wuhan, China). All the measurement of plasma apoCIII and ANGPTL8 were performed in duplicate for each sample. The coefficient of variation for intra- and inter-assay variation was < 6 and < 9%, respectively.

### ApoB-depleted plasma preparation

According to previous reported procedures [11], 540ul heparin sodium solution (280 mg/ml, Aladdin, H104201) and 10 ml manganese chloride solution (1.06 mol/L, Aladdin, M112542) were mixed. 100ul mixed solution was added to 1 ml plasma, incubated for 30 min at 4 °C, followed by centrifugation at 1500 g for 30 min. Supernatant was collected. If supernatant was still turbid (especially samples from patients with hypertriglyceridemia), plasma was centrifuged at 12000 g for 10 min again. Previous study revealed that heparin sodium/manganese chloride precipitation had no effects on HDL size as well as cholesterol efflux measurement [12], and therefore this method was chosen to prepare apoB-depleted plasma in the study.

### Statistical analysis

Statistical analysis was performed with Statistical Package for Social Sciences version 22.0 and plots were made with GraphPad Prism V.6.0 (GraphPad Software, Inc., La Jolla, California, USA). Clinical data are expressed as mean ± standard deviation (normally distributed continuous data) or median with interquartile range (skewed distributed continuous data). Comparisons between categorical data were performed with Chi Squared tests, while continuous variables were assessed by unpaired t test (for normal distribution) or nonparametric test (for skewed distribution). For the variables skewed distributed, logarithmatic-transformed values were used for the analysis. To evaluate the associations between variables, partial correlation analysis was performed. A two tailed *P* value < 0.05 was considered statistically significant.

## Results

### Characteristics of subjects

Demographic and biochemical characteristics of participants are shown in Table 1. The study includes 120 unrelated individuals, 60.80% of the participants were male and the mean age was 64.17 years. Compared to nonCAD controls, CAD subjects had higher free fatty acid (FFA). Besides, the percentage of diabetic subjects and statin users was also significantly higher in CAD group.

**Table 1:** Anthropometric and metabolic characterisitcs of study participants

Variables	All(*n* = 120)	CAD(*n* = 68)	Non-CAD(*n* = 52)	*P* value
Male(%)	60.80	60.29	61.54	NS
Age(years)	64.17 ± 8.11	65.13 ± 8.03	63.91 ± 8.06	NS
BMI(kg/m^2^)	24.43 ± 3.81	24.49 ± 4.57	24.47 ± 3.21	NS
TG(mg/dL)	117.8(87.7–174.5)	120.9(91.5–168.1)	113.4(87.7–182.5)	NS
TC(mg/dL)	155.1(133.0–184.8)	146.6(128.5–183.3)	160.9(133.0–181.0)	NS
HDL-C(mmol/L)	1.09 ± 0.25	1.05 ± 0.25	1.12 ± 0.26	NS
LDL-C(mmol/L)	2.54 ± 0.80	2.40 ± 0.74	2.48 ± 0.82	NS
ApoAI(g/L)	1.13 ± 0.20	1.11 ± 0.19	1.16 ± 0.20	NS
ApoB(g/L)	0.92 ± 0.26	0.89 ± 0.26	0.88 ± 0.27	NS
FFA (mmol/L)	0.48 ± 0.28	0.51 ± 0.29	0.42 ± 0.26	0.026
hsCRP(mg/L)	2.73(1.00–8.98)	2.64(0.71–10.46)	2.30(0.87–4.64)	NS
BUN(mmol/L)	5.91(4.84–7.23)	5.70(4.35–7.30)	6.28(5.32–7.96)	NS
UA(umol/L)	308.1(280.0–405.5)	305.8(283.3–370.9)	341.0(292.1–430.1)	0.054
CR(umol/L)	74.50(57.20–86.30)	77.15(67.68–84.68)	77.90(58.98–98.40)	NS
Diabetes(%)	21.67	32.35	7.69	0.001
Statin use(%)	36.67	54.41	13.46	< 0.0001

Values are expressed as mean ± SD or median (interquartile range). CAD indicates coronary artery disease; *BMI* body mass index, *TG* triglyceride, *TC* total cholesterol, *HDL-C* high density lipoprotein-cholesterol; *LDL-C* low density lipoprotein-cholesterol; *apoAI* apolipoprotein AI, *apoB* apolipoprotein B, *FFA* free fatty acid, *hsCRP* high sensitivity C reactive protein, *BUN* blood urea nitrogen, *UA* uric acid, *CR* creatinine, *apoCIII* apolipoprotein CIII

### ANGPTL8 and ApoCIII concentrations in subjects

**Fig. 1:**
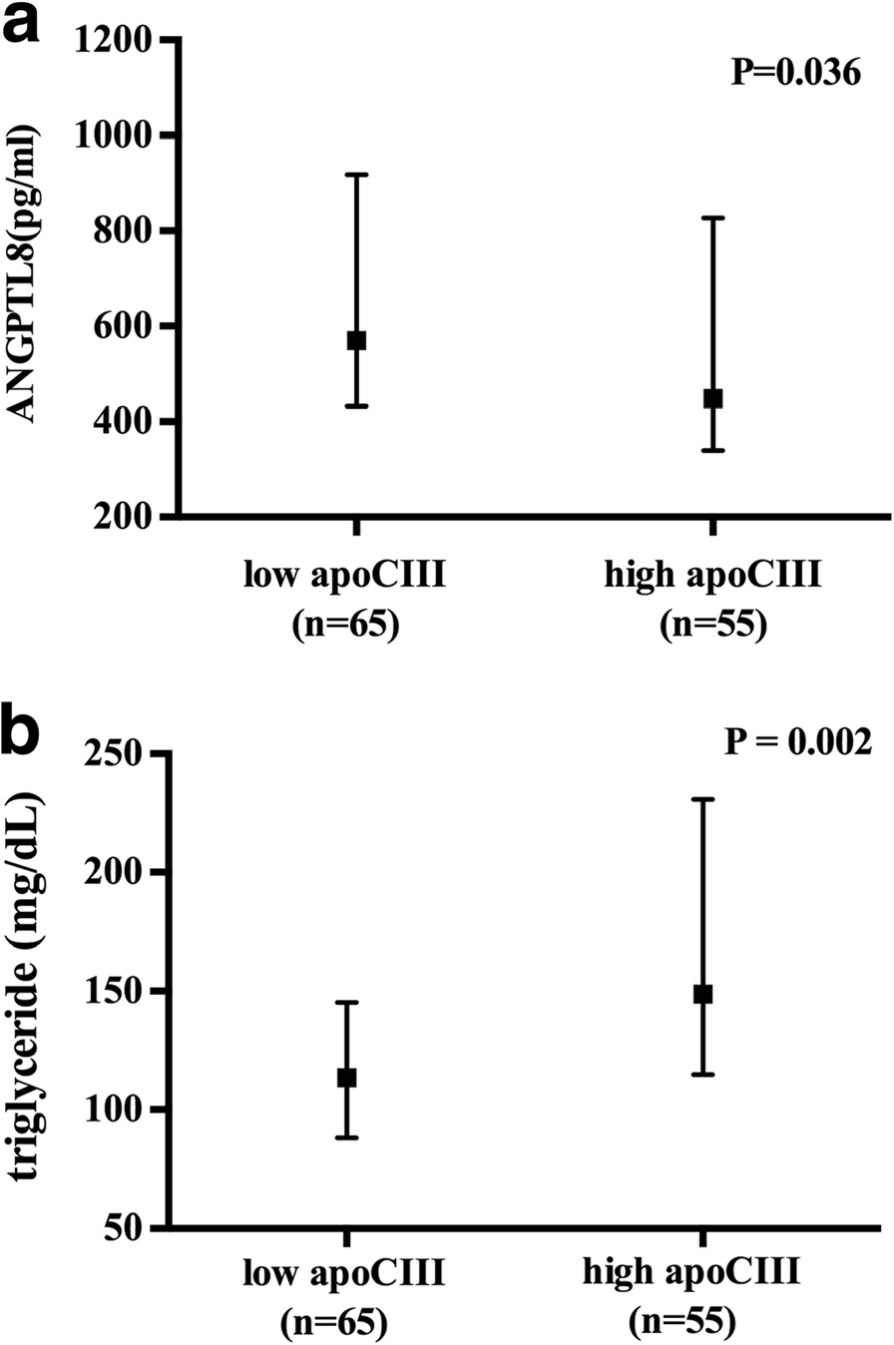
Comparison of circulating ANGPTL8 and apoCIII concentrations under different baseline conditons. Data are expressed as median(interquartile range). **a**. ANGPTL8 concentration in the low apoCIII group and high apoCIII group. **b**. Triglyceride level in the low apoCIII group and high apoCIII group. ApoCIII indicates apolipoprotein CIII; TG, triglyceride; ANGPTL8, angiopoietin-like protein

ApoCIII and ANGPTL8 concentrations were measured in CAD group and nonCAD group (Table 2). Although the differences between these two groups were not significant, the apoCIII_HDL_ concentration and apoCIII_HDL_ ratio was significantly higher in CAD group than that in nonCAD group (Table 2). In addition, subjects were classified according to their apoCIII and ANGPTL8 concentrations (Table 3). Subjects were divided according to their plasma apoCIII level: low apoCIII group (plasma apoCIII < median 11.7 mg/dl) and high apoCIII group (plasma apoCIII ≥ median 11.7 mg/dl). Subjects in the low apoCIII group exhibited significantly higher ANGPTL8 than those in the high apoCIII group [569.6(432.2–917.6) vs 447.8(339.8–827.1), *P* = 0.036, Fig. 1a]. Triglyceride level in the low apoCIII group was significantly lower than that in the high apoCIII group [113.4(88.1–145.3) vs 148.8(114.7–230.7), *P* = 0.002, Fig. 1b].

**Table 2:** Comparison of circulating ANGPTL8 and apoCIII concentrations in CAD group and nonCAD group

	All (n = 120)	CAD (n = 68)	Non-CAD (n = 52)	*P* value
ANGPTL8 (pg/ml)	561.1 (390.1–882.9)	544.4 (374.3–903.7)	597.7 (408.6–853.1)	NS
ApoCIII (mg/dl)	11.7 (9.1–13.5)	10.6 (8.8–12.3)	11.3 (9.0–14.7)	NS
apoCIII_HDL_ (mg/dl)	4.55 (3.32–6.68)	5.01 (3.81–7.44)	3.86 (3.07–6.15)	0.048
apoCIII_HDL_ ratio	0.43 (0.30–0.53)	0.48 (0.31–0.55)	0.37 (0.24–0.47)	0.003

Data are expressed as mean ± SD or median (interquartile range). ANGPTL8 indicates angiopoietin-like protein 8; apoCIII, apolipoprotein CIII; apoCIII_HDL_, apolipoprotein CIII in apoB-depleted plasma; apoCIII_HDL_ ratio, apolipoprotein CIII in apoB-depleted plasma over plasma apolipoprotein CIII; CAD, coronary artery disease

**Table 3:** Comparison of circulating ANGPTL8 and apoCIII concentrations under different baseline conditions

	Low apoCIII (*n* = 65)	High apoCIII (*n* = 55)	*P* value
ApoCIII(mg/dl)	/	/	/
apoCIII_HDL_(mg/dl)	3.9 (3.1–5.1)	5.0 (3.4–8.2)	0.014
apoCIII_HDL_ ratio	0.44 (0.36–0.53)	0.34 (0.23–0.51)	0.052
ANGPTL8(pg/ml)	569.6 (432.2–917.6)	447.8 (339.8–827.1)	0.036
Triglyceride(mg/dl)	113.4 (88.1–145.3)	148.8 (114.7–230.7)	0.002
CAD percentage	66.2%	43.2%	0.037

Data are expressed as mean ± SD or median (interquartile range). ANGPTL8 indicates angiopoietin-like protein 8; apoCIII, apolipoprotein CIII; apoCIII_HDL_, apolipoprotein CIII in apoB-depleted plasma; apoCIII_HDL_ ratio, apolipoprotein CIII in apoB-depleted plasma over plasma apolipoprotein CIII; CAD, coronary artery disease

### Correlation analysis of clinical variables with ANGPTL8 and apoCIII

**Fig. 2:**
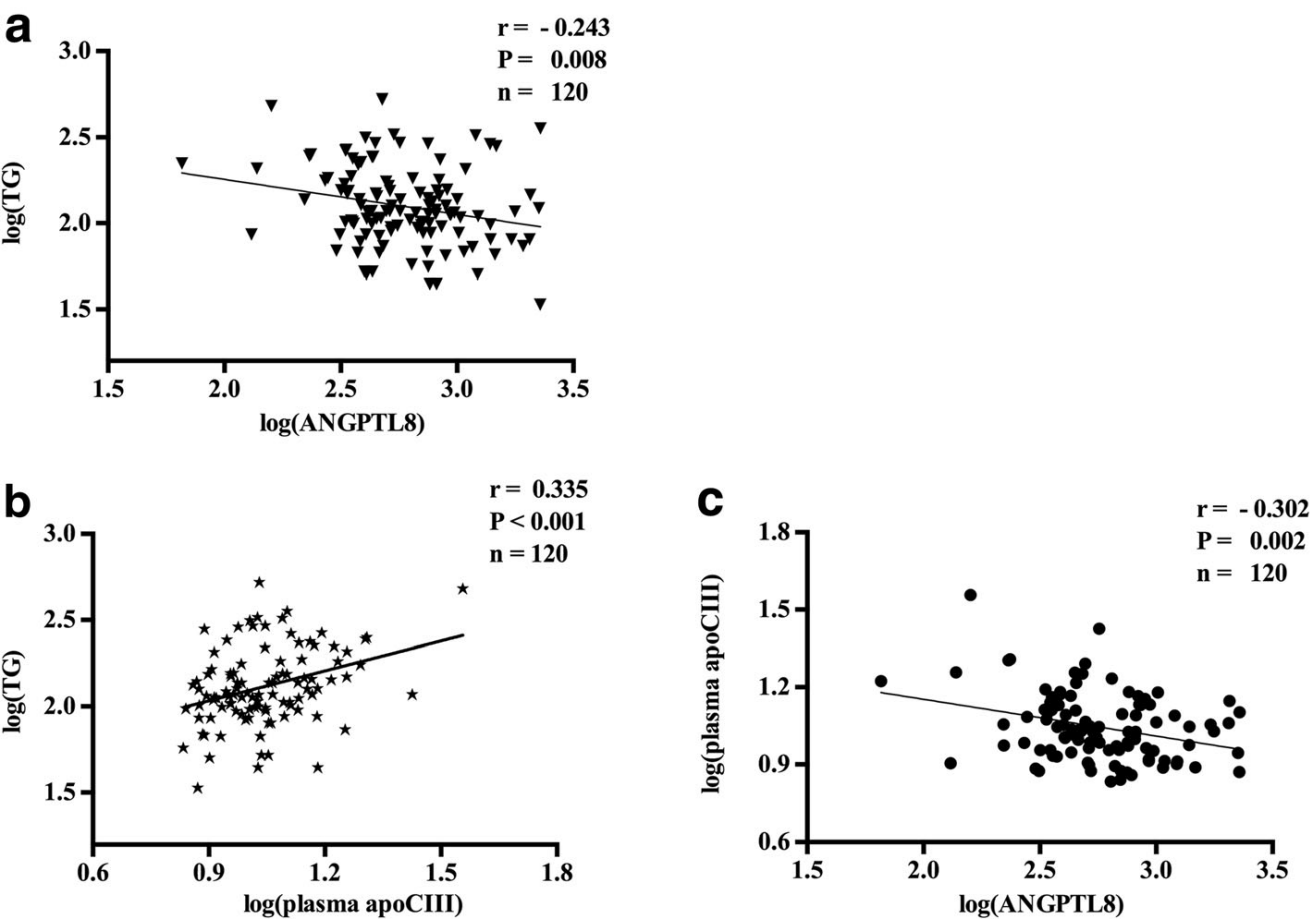
Pearson’s correlations between clinical variables and log-transformed ANGPTL8 as well as log-transformed apoCIII in all the subjects. **a**. ANGPTL8 and TG. **b**. ApoCIII and TG. **c**. ANGPTL8 and apoCIII. TG indicates triglyceride; ANGPTL8, angiopoietin-like protein; apoCIII, apolipoprotein CIII

To investigate variables associated with ANGPTL8 and apoCIII, correlation analysis was performed. Correlation coefficients between clinical variables and plasma ANGPTL8 as well as apoCIII concentration (both log-transformed) were present in Table 4. The results showed that ANGPTL8 was positively correlated with age (*r* = 0.240, *P* = 0.008), but inversely correlated with triglyceride (*r* = − 0.243, P = 0.008, Fig. 2a), and renal function biomarkers including BUN (*r* = 0.351, *P* < 0.0001), UA (*r* = 0.333, *P* < 0.001) and CR (*r* = 0.509, *P* < 0.0001). On the other hand, plasma apoCIII presented a positive relationship with triglyceride (*r* = 0.335, *P* < 0.001, Fig. 2b). Besides, ANGPTL8 was inversely correlated with plasma apoCIII (*r* = − 0.302, *P* = 0.002, Fig. 2c).

**Table 4:** Pearson’s correlations between clinical variables and log-transformed ANGPTL8 as well as log-transformed apoCIII in all the subjects

	ANGPTL8		Plasma apoCIII		apoCIII_HDL_		apoCIII_HDL_ ratio	
	r	*P* value	r	*P* value	r	*P* value	r	*P* value
Age	0.240	0.008	−0.075	0.456	0.102	0.270	0.042	0.677
BMI	0.075	0.417	−0.033	0.740	−0.025	0.783	−0.061	0.542
CR(log-transformed)	0.509	< 0.0001	0.012	0.909	0.120	0.196	0.096	0.342
BUN(log-transformed)	0.351	< 0.0001	−0.107	0.291	0.064	0.494	0.050	0.623
UA(log-transformed)	0.333	< 0.001	0.131	0.194	0.179	0.053	−0.035	0.731
TG(log-transformed)	−0.243	0.008	0.335	< 0.001	−0.241	0.008	−0.316	0.001
TC(log-transformed)	−0.245	0.007	0.198	0.046	−0.102	0.270	−0.012	0.904
HDL-C	−0.002	0.975	−0.059	0.553	0.135	0.144	0.302	0.002
LDL-C	−0.224	0.014	0.165	0.097	−0.069	0.453	−0.020	0.845
apoAI	−0.102	0.270	0.108	0.283	−0.014	0.877	0.117	0.245
apoB	− 0.167	0.069	0.208	0.037	−0.104	0.263	−0.064	0.522
hsCRP(log-transformed)	0.067	0.471	0.067	0.505	0.131	0.156	−0.013	0.901
ANGPTL8(log-transformed)	/	/	−0.302	0.002	0.089	0.331	0.158	0.113
ApoCIII(log-transformed)	−0.302	0.002	/	/	0.319	0.001	−0.217	0.029
apoCIII_HDL_(log-transformed)	0.089	0.331			/	/	0.796	< 0.0001
apoCIII_HDL_ ratio	0.158	0.113			0.796	< 0.0001	/	/

*BMI* indicates body mass index, *CR* creatinine, *BUN* blood urea nitrogen *UA* uric acid, *TG* triglyceride, *TC* total cholesterol, *HDL-C* high density lipoprotein-cholesterol, *LDL-C*, low density lipoprotein-cholesterol, *apoAI* apolipoprotein AI, *apoB* apolipoprotein B, *hsCRP* high-sensitivity C reactive protein, *ANGPTL8* angiopoietin-like protein 8, *apoCIII* apolipoprotein CIII, *apoCIII*_*HDL*_ apolipoprotein CIII in apoB-depleted plasma, *apoCIII*_*HDL*_ *ratio* apolipoprotein CIII in apoB-depleted plasma over plasma apolipoprotein CIII

### Multivariate analysis for the associations of clinical variables to triglyceride

In order to determine the independent contributors to triglyceride, stepwise multiple regression models were fitted after adjustment for different variables (Table 5). Log-transformed values were used for the variables skewed distributed, including ANGPTL8 and apoCIII. In addition, plasma apoCIII level was an independent contributor to the triglyceride level. When apoCIII was introduced into the regression model (model 3), the relationship between ANGPTL8 and triglyceride level disappeared.

**Table 5:** Independent contributors to the triglyceride level

	R square	β	Standardized β	*P* value
Model 1	0.207			< 0.001
Age		−0.005	−0.171	0.056
HDL-C		−0.269	−0.308	< 0.001
ANGPTL8 (log-transformed)		−0.170	−0.203	0.020
Model 2	0.234			< 0.001
Age		−0.005	−0.189	0.028
HDL-C		−0.265	−0.303	< 0.001
ApoCIII (log-transformed)		0.153	0.259	0.002
Model 3	0.257			< 0.001
Age		−0.004	−0.153	0.079
HDL-C		−0.273	−0.312	< 0.001
ANGPTL8 (log-transformed)		−0.133	−0.159	0.064
ApoCIII (log-transformed)		0.136	0.230	0.006

*HDL-C* indicates high density lipoprotein-cholesterol, *ANGPTL8* angiopoietin-like protein 8; *apoCIII* apolipoprotein CIII

## Discussion

In this study, we found that ANGPTL8 and apoCIII were significantly correlated with triglyceride level. Besides, stepwise multiple regression analysis revealed that apoCIII was an independent contributor to triglyceride level.

Many clinical studies and animal experiments have shown that ANGPTL8 was highly involved in triglyceride metabolism. ANGPTL8 knockout mice presented nearly 70% reduced plasma triglyceride levels compared to the wild-type controls after feeding [13], while ANGPTL8 overexpression significantly increased plasma triglyceride levels by five folds [14]. ANGPTL8 was found to inhibit LPL activity and disrupt triglyceride clearance partly via ANGPTL3 activation [4]. In the cohort of Beijing children and adolescents metabolic syndrome (BCAMS) study, participants with high TG (defined as ≥150 mg/dl) exhibited significantly increased ANGPTL8 concentration [15]. However, our experiments showed that ANGPTL8 was significantly higher in the low TG group when subjects were divided according to their TG levels. Another study that aimed at dyslipidemic middle-aged cohorts in Caucasian population also found that subjects with lower TG displayed significantly higher ANGPTL8 levels than subjects with higher TG (defined as ≥150 mg/dl) [16]. The disparity among these population studies might be caused by the sample selection. Young population (20.2 ± 2.9 years old) with risk for metabolic syndrome was selected in BCAMS study [15], while older patients were recruited in the current study (64.17 ± 8.11 years old) and the study aimed at Caucasian middle-aged population study [16]. Besides, ANGPTL8 was also positively correlated with age, and therefore the relationship between triglyceride and ANGPTL8 might be confounded by age.

The physiological regulation of LPL activity is driven via post-translational mechanisms including ANGPTLs (ANGPTL 3, 4 and 8) and apolipoproteins (apoCIII and apoAV) [5]. In circulation, apoCIII, mainly residing on the surface of HDL and TRLs, inhibited LPL activity and disrupted TRLs clearance [7], thereby leading to hypertriglyceridemia. In this study, plasma apoCIII exhibited a relatively strong correlation with triglyceride. Besides, the relationship between ANGPTL8 and triglyceride disappeared when apoCIII was introduced into the regression model (model 3), suggesting that the effect of ANGPTL8 on triglyceride metabolism might be apoCIII dependent. Previous study found that ANGPTL8 regulated LPL activity and triglyceride metabolism partly dependent on ANGPTL3, but other factors beyond ANGPTL3 might also mediate the effects of ANGPTL8 on triglyceride regulation [4]. Our research provided a hint that apoCIII might be involved in ANGPTL8 modulation of LPL activity and triglyceride metabolism. However, whether the interaction exists awaits further investigation.

Interestingly, ANGPTL8 was strongly correlated with biomarkers of renal function (BUN, UA and CR). Although the relationship between ANGPTL8 and eGFR was still controversial [17, 18], a recent study conducted on T2DM patients revealed that ANGPTL8 was associated with urinary albumin excretion and renal function [18]. In addition, the study also showed that ANGPTL8 could increase the risk of diabetic nephropathy (DN) and might serve as a predictor for DN progression [18]. Kidney may be the important organ for ANGPTL8 degradation and excretion. When ANGPTL8 could not be efficiently cleaned, accumulated ANGPTL8 might cause dysregulated lipids metabolism in the kidney, leading to lipids accumulation in the artery wall, foam cells formation, atherosclerosis deterioration and glomerulosclerosis occurrence [19].

We acknowledged the limitations of our study. First, the cross-sectional nature of our study did not provide the direct proof for the causality. Additionally, we only measured ANGPTL8 in the fasting state, but ANGPTL8 activity was regulated by nutritional status [14]. Therefore the association of ANGPTL8 and apoCIII in the postprandial state still needed to be validated in the future research. Finally, we measured ANGPTL8 with EIAAB ELISA kits that recognizes N-terminus and measures the full-length form of ANGPTL8. Although our research presented similar ANGPTL8 levels with previous studies adopting the same kind of ELISA kit, the adoption of different ELISA kits which measured different forms of ANGPTL8 might cause some discrepancies in the results [20].

## Conclusions

In conclusion, our results showed that circulating ANGPTL8 presented a strong relationship with biomarkers of renal function, including BUN, UA and CR. Moreover, ANGPTL8 was inversely correlated with plasma apoCIII and the association between ANGPTL8 and triglyceride disappeared when plasma apoCIII was taken into consideration, suggesting the potential role of plasma apoCIII in ANGPTL8 action. Further research is warranted to elucidate the relationship between apoCIII and ANGPTL8 and the underlying mechanism of triglyceride regulation.

## Abbreviations

ANGPTL8: Angiopoietin-like protein 8
apoCIII: apolipoprotein CIII
CAD: coronary artery disease
DN: diabetic nephropathy
HDL: high-density lipoprotein
LDL-C: low-density lipoprotein cholesterol
LPL: lipoprotein lipase
NAFLD: non-alcoholic fatty liver disease
RIFL: refeeding-induced in fat and liver
TRLs: triglyceride-rich lipoproteins
